# Region-Based Partial Volume Correction Techniques for PET Imaging: Sinogram Implementation and Robustness

**DOI:** 10.1155/2013/435959

**Published:** 2013-12-17

**Authors:** Mike Sattarivand, Jennifer Armstrong, Gregory M. Szilagyi, Maggie Kusano, Ian Poon, Curtis Caldwell

**Affiliations:** ^1^Department of Medical Biophysics, University of Toronto, Odette Cancer Centre at Sunnybrook Health Sciences Centre, Room TG-217, 2075 Bayview Avenue, Toronto, ON, Canada M4N 3M5; ^2^Department of Electrical and Computer Engineering, University of McMaster, 1280 Main Street West, Hamilton, ON, Canada L8S 4K1; ^3^L.C. Campbell Cognitive Neurology Research Unit, Sunnybrook Health Sciences Centre, 2075 Bayview Avenue, Toronto, ON, Canada M4N 3M5; ^4^Institute of Medical Science, 1 King's College Circle, University of Toronto, Toronto, ON, Canada M5S 1A8; ^5^Department of Medical Physics, Odette Cancer Centre at Sunnybrook Health Sciences Centre, 2075 Bayview Avenue, Toronto, ON, Canada M4N 3M5; ^6^Department of Radiation Oncology, University of Toronto, Faculty of Medicine, 150 College Street, Room 106, Toronto, ON, Canada M5S 3E2; ^7^Department of Radiation Oncology, Odette Cancer Centre at Sunnybrook Health Sciences Centre, 2075 Bayview Avenue, Toronto, ON, Canada M4N 3M5; ^8^Department of Medical Imaging, University of Toronto, Sunnybrook Health Sciences Centre, 2075 Bayview Avenue, Toronto, ON, Canada M4N 3M5

## Abstract

*Background/Purpose*. Limited spatial resolution of positron emission tomography (PET) requires partial volume correction (PVC). Region-based PVC methods are based on geometric transfer matrix implemented either in image-space (GTM) or sinogram-space (GTMo), both with similar performance. Although GTMo is slower, it more closely simulates the 3D PET image acquisition, accounts for local variations of point spread function, and can be implemented for iterative reconstructions. A recent image-based symmetric GTM (sGTM) has shown improvement in noise characteristics and robustness to misregistration over GTM. This study implements the sGTM method in sinogram space (sGTMo), validates it, and evaluates its performance. *Methods*. Two 3D sphere and brain digital phantoms and a physical sphere phantom were used. All four region-based PVC methods (GTM, GTMo, sGTM, and sGTMo) were implemented and their performance was evaluated. *Results*. All four PVC methods had similar accuracies. Both noise propagation and robustness of the sGTMo method were similar to those of sGTM method while they were better than those of GTMo method especially for smaller objects. *Conclusion*. The sGTMo was implemented and validated. The performance of the sGTMo in terms of noise characteristics and robustness to misregistration is similar to that of the sGTM method and improved compared to the GTMo method.

## 1. Introduction

In spite of continuous improvement in the instrumentation of positron emission tomography (PET), its spatial resolution still remains relatively low compared to anatomical imaging modalities such as magnetic resonance (MR) or computed tomography (CT). Failure to implement a partial volume correction (PVC) in quantitative PET imaging may result in significant bias in the estimate of regional radioactivity uptake [1–3]. The limited spatial resolution of PET is due to several factors that influence the image formation processes, including positron range, noncollinearity, detector width, and reconstruction filtering [4]. Two distinct effects are usually associated with the partial volume effect [5]. The first is the point response effect, which causes spillover between different regions. This effect can be accounted for with a knowledge of the three-dimensional (3D) PET image formation processes or a measurement of the global PET point spread function (PSF). Usually, a fitted 3D Gaussian curve characterized by its full width half maximums (FWHMs) in the *x*, *y*, and *z* directions is used to estimate the global PET PSF. The second effect is the tissue fraction effect due to the coarse spatial sampling of the PET images, which may cause a single PET voxel to contain more than one tissue type with different tracer uptakes. With the availability of anatomical images in current PET-CT (or PET-MR) systems, this effect can be accounted for by segmented coregistered CT (or MR) images.

Two general categories for PVC implementations are voxel-based and region-based approaches [5]. Some voxel-based techniques require anatomical CT (or MR) co-registered images [6–9] and others in this category do not [10–12]. Voxel-based techniques most often do not require CT (or MR) image segmentation, which could be considered appealing in some applications. Nevertheless, since the correction is performed at the voxel level, the main disadvantage of these techniques is the noise amplification [5] which could be a limiting factor. A region containing many voxels is less likely to be affected by image noise compared to a single voxel.

Region-based PVC approaches are mainly based on a key study by Rousset et al. [13], in what is commonly referred to as the geometric transfer matrix (GTM) method. In this method, the CT (or MR) images are segmented to different nonoverlapping regions representing different tissue types. Using these regions, the GTM method corrects for both the PET tissue fraction effect and the point response effect in an analytical approach. The GTM method is attractive since it is straightforward to implement and it provides meaningful physical interpretations for spillover between all regions. The GTM method indeed remains the most widely used PVC method [12] and is usually considered as the reference PVC method [10, 14]. Often new PVC methods, even those that are nonregion-based, are evaluated in performance against the GTM method [10, 12, 14, 15].

The GTM method obtains spillover information between regions by calculating regional spread functions (RSFs). Two distinct approaches to calculate RSFs have been used in previous studies, which are very different in terms of computational needs. The first approach forward projects each region to “sinogram-space” and sinograms are then reconstructed to obtain RSFs [16, 17]. This approach requires that the PET detector geometry be known and was used in the original GTM method [13], where 3D resolution effects were modeled using an analytical simulator of a 2D-acquisition PET system [18]. The second approach, which is used more commonly, is to convolve the regions directly in “image-space” with a global PSF [19–22]. Throughout this paper, we refer to the image-based approach as the GTM method and the sinogram-based approach as the GTMo method. The “o” here refers to the letter “o” in “sinogram.”

A study by Frouin et al. [23] compared the GTM and GTMo methods by implementing them for data acquired using a 3D-acquisition PET system, an acquisition mode that most current PET systems are based on. Although the GTMo method is computationally slower than the GTM method, the GTMo method more closely simulates the 3D image formation and acquisition processes of a physical PET system compared to the GTM method which uses a global 3D PSF. Thus, the GTMo method automatically accounts for spatial variations in the PET PSF [5]. Moreover, only a sinogram-based method can be readily extended to the iterative reconstruction algorithms [17] which are now more commonly used in the clinic. The 3D implementation of GTMo by Frouin was adapted by some studies for clinical applications [24].

While accurate regional recovery is the main goal of any PVC, precision and noise propagation are important considerations in evaluating the performance of any PVC method. Performance can be further evaluated in terms of robustness to PET-CT (or PET-MR) misregistration, as well as with regard to errors in PET PSF measurements. The study by [23] showed that the performance of the GTMo method is similar to that of the GTM method in terms of accuracy, precision, and robustness with regard to registration errors. Robustness to PET-CT (or PET-MR) registration is of special concern if patient motion exists between the two scans. Both the GTM and GTMo methods are especially vulnerable to registration errors [23]. Misregistration has been reported [14] to be the factor with the largest impact on the accuracy and precision of the GTM method.

Recently, a new region-based PVC has been reported [25] which is referred to as the symmetric geometric transfer matrix (sGTM) method. The accuracy of the sGTM method is similar to that of GTM, while it has better characteristics for noisy PET images in terms of precision and noise propagation. The sGTM method was also reported to be more robust than the GTM method, both in terms of registration errors and errors in PSF measurements. Similar to GTM, the sGTM method is attractive in a sense that it provides an analytical equation with meaningful physical interpretations for spillover between all regions. The implementation of sGTM does not incur any additional computational cost compared to the GTM method.

The sGTM method referred to above was implemented in image-space, similar to the GTM method. No previous study has implemented the sGTM method in sinogram-space. As mentioned previously, a sinogram implementation is of interest since it more closely simulates 3D PET image formation processes compared to the image-space implementation and it provides the other advantages noted above. The objective of this study was to implement and validate the sGTM method for 3D PET in sinogram-space. In this study we refer to this implementation as the sGTMo method. This study also compares the performance of the sGTMo method to previous region-based PVC methods, that is, GTMo and sGTM. Two hypotheses are tested in this comparison: (a) just as GTMo was reported to be similar in performance to the GTM method [23], the first hypothesis is that the sGTMo method performs similarly to the sGTM method and (b) since the sGTM was shown to have performance advantages over the GTM method [25], the second hypothesis is that the sGTMo method would show similar performance advantages over the GTMo method.

## 2. Materials and Methods

### 2.1. Principles of Region-Based PVC Methods

The principles of region-based PVC methods are described in the literature [13, 25]. In this section, the implementation of four different region-based PVC methods is described, that is, GTM, GTMo, sGTM, and sGTMo, that were used to obtain the results in this paper.

In short, high resolution CT (or MR) images are segmented into *N* tissue types with nonoverlapping volumes of interest VOI_*i*_  (*i* = 1 ⋯ *N*). Uptake for each tissue type is assumed to be homogenous and the goal is to obtain this uptake value with knowledge of the PET 3D PSF or the PET 3D image formation process. The mask image for each VOI contains binary voxel values of unity (if the voxel belongs to the tissue) or zero (if the voxel is outside the tissue). The *N* VOI masks are each interpolated from the CT (or MR) voxel size to the coarser voxel size of PET to account for the tissue fraction effect and obtain PET-space VOI images.

In the GTM and sGTM methods, each PET-space VOI image is convolved in 3D with the PET PSF to obtain the RSFs, which are blurred versions of the corresponding tissue masks. In the GTMo and sGTMo methods, the global PET PSF is not used, and in order to obtain the RSFs, three steps are required. First, the PET-space VOI images are forward projected with knowledge of the 3D PET scanner acquisition geometry. The resulting projection sinograms containing 3D line of responses (LORs) are then blurred. Finally, the projection sinogram for each VOI is reconstructed with a 3D reconstruction algorithm to obtain the corresponding RSF. These three steps, in effect, simulate the PET image formation process for each tissue volume separately. We implemented the first and last steps using the open source Software for Tomographic Image Reconstruction (STIR), details of which are described in Section 2.4.2 below. The middle step (blurring) may be performed by convolving each LOR with the intrinsic PSF of 2 crystals [23]. We implemented this step in the Fourier domain by multiplying the Fourier transform (FT) of the sinograms with a Gaussian function such that the resulting 3D PSF from a simulated point source matched the measured PSF of the PET scanner.

Note that the RSF images are different for the image-based PVC methods (GTM and sGTM) versus the sinogram-based PVC methods (GTMo or sGTMo) due to essentially different methods of calculation that account for resolution loss in PET.

To perform a PVC, all four methods use the following equation:

(1) $$\begin{array}{c} {\left[\omega_{ij}\right]}_{N\times N}{\left[T_{j}\right]}_{N\times 1}={\left[t_{j}\right]}_{N\times 1}, \end{array}$$

where in GTM and GTMo methods [13],

(2) $$\begin{array}{c} \omega_{ij}=\overset{}{\underset{\text{FOV}}{\int }}\text{RS}{\text{F}}_{i}\left(r\right)\cdot \text{VO}{\text{I}}_{j}\left(r\right)dr,\\ t_{j}=\overset{}{\underset{\text{FOV}}{\int }}I\left(r\right)\cdot \text{VO}{\text{I}}_{j}\left(r\right)dr \end{array}$$

and in sGTM and sGTMo methods [25],

(3) $$\begin{array}{c} \omega_{ij}=\overset{}{\underset{\text{FOV}}{\int }}\text{RS}{\text{F}}_{i}\left(r\right)\cdot \text{RS}{\text{F}}_{j}\left(r\right)dr,\\ t_{j}=\overset{}{\underset{\text{FOV}}{\int }}I\left(r\right)\cdot \text{RS}{\text{F}}_{j}\left(r\right)dr. \end{array}$$

Here, *I*(*r*) is the measured PET image with a given field of view (FOV) and [*ω*_*ij*_]_*N*×*N*_ is commonly referred to as the geometric transfer matrix which contains weighing factors with a physical interpretation for each element. In the GTM and GTMo methods, *ω*_*ij*_ describes spillover from one VOI mask to another. In the sGTM and sGTMo methods, on the other hand, this matrix is symmetric and *ω*_*ij*_ describes spillover from one RSF to another. [*t*_*j*_]_*N*×1_ on the right side of the equation is where we sample the measured PET image for each region. In GTM and GTMo methods, this is performed by multiplying the PET image by the corresponding VOI mask, while in the sGTM and sGTMo methods, this task is performed by multiplying the PET image by the corresponding RSF image. [*T*_*j*_]_*N*×1_ is a vector containing estimates of true uptake values for each tissue type and in order to apply PVC, (1) can be solved by multiplying both sides of the equation by the inverse of [*ω*_*ij*_]_*N*×*N*_.

**Figure 1:**
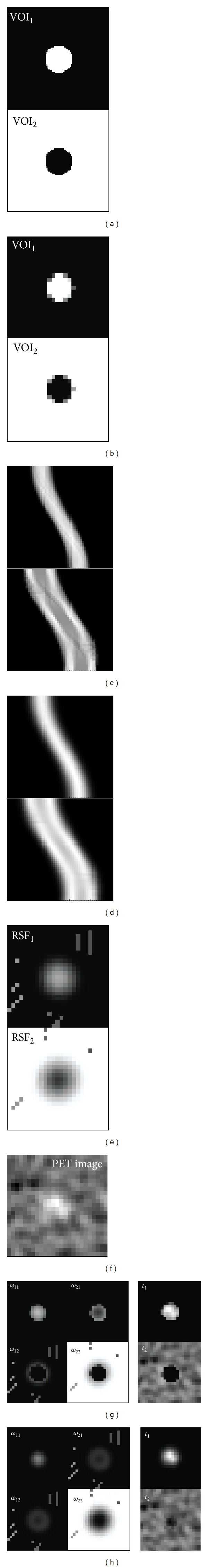
High resolution CT (or MR) image of a vessel is segmented to create two VOI masks (a). These VOI masks are then interpolated to the coarser PET voxel size to account for the tissue fraction effect (b). Note that, unlike VOI masks in (a), voxel values in (b) may have values between zero and one in the boundary region. The PET-space VOI image is then forward projected using the 3D PET scanner geometry (c). The projection image is blurred in sinogram-space (d). The sinograms are then reconstructed with a 3D PET reconstruction algorithm to obtain RSF images (e). With a given measured PET image (f) and calculated RSF images, parameters of (1) can be obtained for GTMo (g) or sGTMo (h) PVC methods.

Figure 1 illustrates the steps of implementing PVC in sinogram-space (GTMo and sGTMo) for an image of a vessel having only two tissue types (*N* = 2). Note that unlike RSFs obtained by convolution in GTM and sGTM, where voxel values are only between zero and one, RSFs obtained for GTMo and sGTMo may contain negative voxel values and streak artifacts as shown in Figure 1(e). This effect is the result of the reconstruction process and may translate into the weighting matrices as shown in Figures 1(g) and 1(h). However, this effect does not pose a problem to the calculation of weighting factors. This can be confirmed by adding all the RSF images and verifying that all the voxels are close to unity [25]:

(4) $$\begin{array}{c} \overset{N}{\underset{i=1}{\sum }}\text{RS}{\text{F}}_{i}\left(r\right)=1. \end{array}$$

For the data presented in this paper, the deviations from unity were less than 0.5% for all voxels and thus we ignored this effect.

The performances of the four region-based PVC methods were evaluated by implementing them for two 3D simulated phantoms (a sphere phantom and a brain phantom) and one physical sphere phantom.

### 2.2. Simulations

#### 2.2.1. Simulation of 3D Sphere Phantom

**Figure 2:**
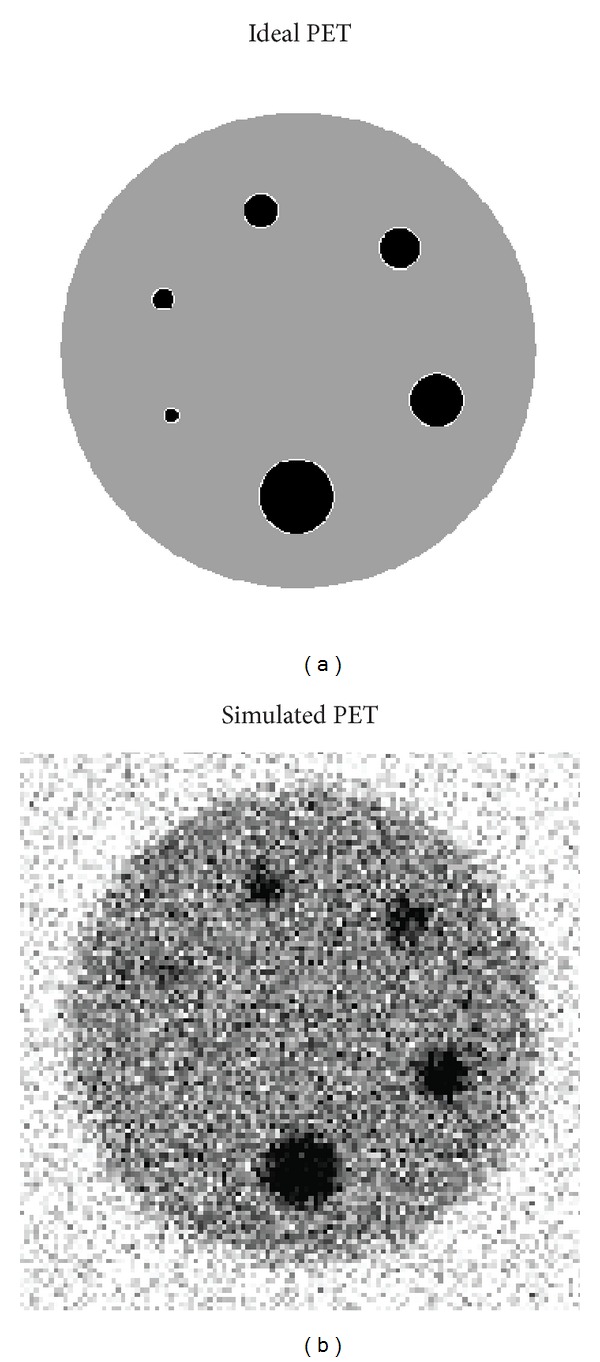
A transaxial slice of an ideal 3D PET image (in CT-space) is shown in (a) which was created from the physical sphere phantom in Figure 4(a). A transaxial slice of the simulated 3D PET image is shown in (b) which was created for the GTMo and sGTM PVC methods.

A 3D sphere PET phantom was simulated using the geometry obtained from a CT scan of the physical sphere phantom shown in Figure 4(a). The spheres were fixed in a cylindrical tank and their sizes ranged in inner diameter from 5 to 30 mm and the wall thickness for all spheres was 0.6 mm. The CT voxels were isotropic, 0.6 mm on each side. The CT images of the phantom were contoured automatically using an in-house analytic sphere segmentation algorithm to obtain the location of the spheres and the outer walls relative to the tank. Using the CT geometry and voxel size, an ideal PET image was obtained by assigning the relative uptake of the sphere-to-background ratio of 3 to 1 and the wall uptake to zero as shown in Figure 2(a). This CT-space ideal PET image was then downsampled using a trilinear interpolation algorithm to the PET voxel size to account for the tissue fraction effect. The PET voxel size was 2 × 2 × 3.15 mm^3^ in the *x*, *y*, and *z* directions, respectively, the same as that of the physical phantom PET scan. For the GTM and sGTM PVC methods the ideal PET image was then convolved in 3D with the PET PSF and noise was added to obtain the simulation PET image. The PSF was Gaussian with FWHMs of 7.23, 7.14, and 6.65 mm in the *x*, *y*, and *z* directions, respectively, matching the PSF for the physical sphere phantom. For the GTMo and sGTMo PVC methods the ideal PET image was forward projected, filtered, and reconstructed using the 3D filtered back projection (FBP) algorithm, and noise was added to create a simulated PET image as shown in Figure 2(b). The forward projection and reconstruction parameters were chosen to match those of the physical sphere phantom. The details of these parameters are described in Section 2.4.2 below. The added noise was uniform across the phantom and uncorrelated for all four PVC methods and had a Gaussian distribution with a standard deviation of 25% relative to the mean tank uptake. This noise level was chosen to match the voxel noise obtained from the physical phantom. A total of 100 3D PET images were simulated, each with different stochastic noise and the four PVC methods were applied to each 3D PET image. For all PVC methods, three VOIs were chosen, that is, inner sphere volume, sphere wall, and the background volume, which resulted in 3 × 3 weighting matrices. The background volume for each sphere size was a cylinder in the tank around the sphere such that its dimensions were at least 20 mm larger than the outer sphere walls in all directions. The geometry and the parameters (reconstruction, noise, etc.) of the simulated PET sphere phantom were chosen to be identical to those of the physical PET phantom. This approach provides an opportunity to directly compare the results for the simulated and the physical sphere phantoms.

#### 2.2.2. Simulation of 3D Brain Phantom

**Figure 3:**
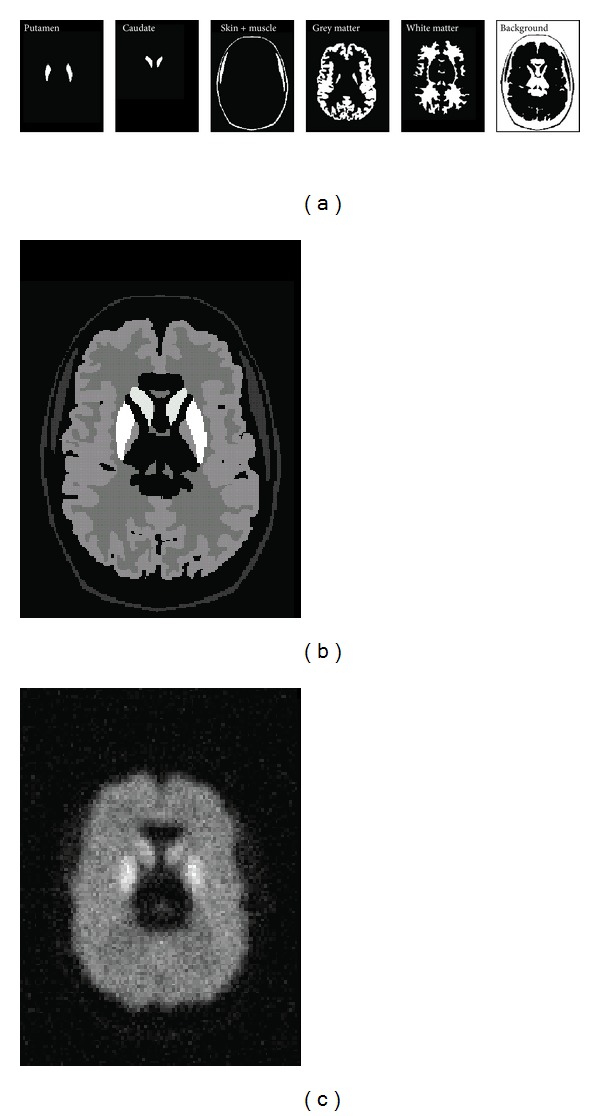
Five VOIs plus background (a) were chosen from the Zubal brain phantom. These VOIs were assigned different relative uptake values to create an ideal PET image in MR-space (b). The ideal PET image was then downsampled, forward projected, filtered, and reconstructed to create a simulated PET image (c) for GTMo and sGTMo PVC methods.

A 3D PET brain phantom was simulated from the segmented images of a dedicated MRI head phantom made available by techniques described in a previous study [26]. Five VOIs were chosen from the Zubal phantom: right and left putamen, right and left caudate, skin and skeletal muscle, grey matter, and white matter. The choice of tissue volumes is typically based on the research question involved and the characteristics of the tracer used. The VOIs for the brain phantom in this study are those of a previous study [23] for striatal brain PET imaging using 18F-L-dopa where small VOIs (e.g., putamen and caudate) are involved in the PVC. The five VOIs were assigned relative uptake values of 4.5, 4.0, 1.0, 2.5, and 2.0, respectively. These uptake values were taken from [23] for the 18F-L-dopa tracer. The rest of the image volume was assigned to be the background VOI with a relative uptake of zero. Thus a total of six VOIs were assigned to create 6 × 6 weighting matrices for all four PVC methods. These six VOIs are shown in Figure 3(a). The ideal PET image in MR-space after assigning the uptake values is shown in Figure 3(b). The MR voxel size was 1.1 × 1.1 × 1.4 mm in the *x*, *y*, and *z* directions, respectively. The ideal 3D MR-space image was then down-sampled to match the voxel size of the PET image to account for the tissue fraction effect using a tri-linear interpolation algorithm. The PET voxel size was 2 × 2 × 3.15 mm^3^ in the *x*, *y*, and *z* directions, respectively. For the GTM and sGTM PVC methods, the down-sampled PET image was then convolved in 3D with the PET PSF and noise was added to create simulated 3D brain PET images. The Gaussian 3D PSF had FWHM values of 7.23, 7.14, and 6.65 mm in the *x*, *y*, and *z* directions, respectively. For the GTMo and sGTMo PVC methods the down-sampled PET image was forward projected, filtered, and reconstructed using the 3D FBP algorithm and then noise was added to create simulated 3D brain PET images.  Figure 3(c) shows the simulated PET image for the GTMo and sGTMo PVC methods. Similar to the simulated sphere phantom, a 25% uncorrelated voxel noise was added uniformly for all four PVC methods. A total of 100 3D PET images were created each with different stochastic noise and the four PVC methods were applied to each 3D image.

### 2.3. Physical Sphere Phantom

**Figure 4:**
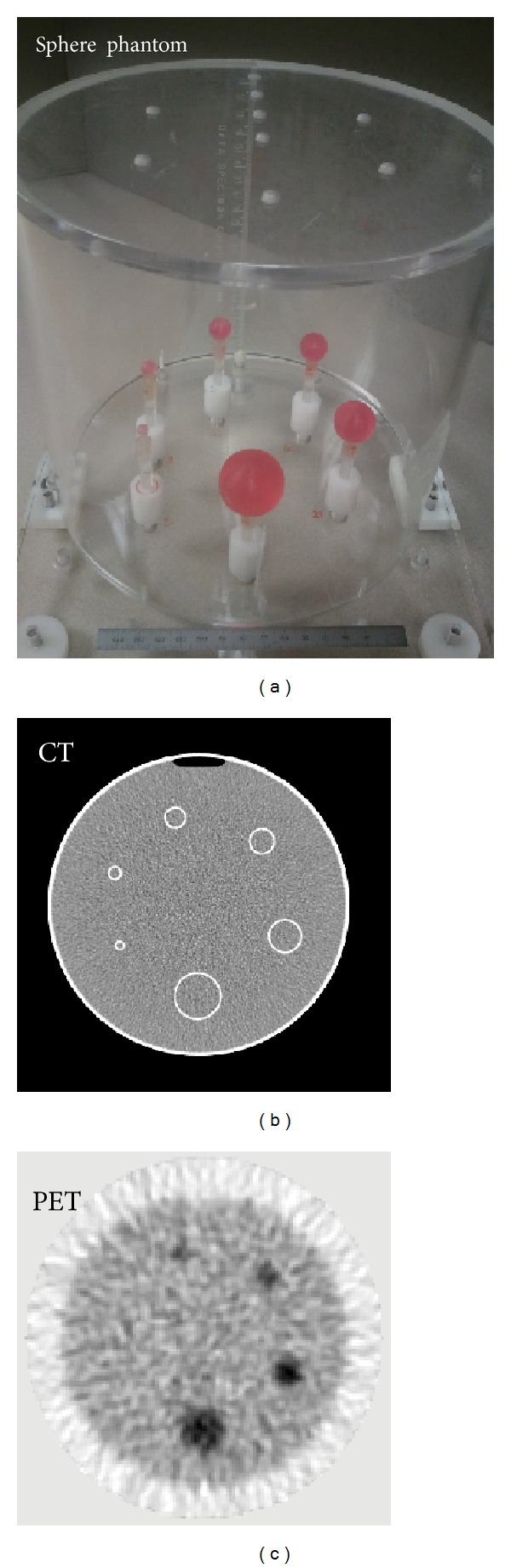
The physical sphere phantom (a) and a transaxial slice of CT (b) and PET (c) images of it.

A physical sphere phantom shown in Figure 4(a) was constructed that had six fillable spheres with inner diameters ranging from 5 to 30 mm and a wall thickness of 0.6 mm for all the spheres. The spheres were fixed in a cylindrical tank with a diameter of 20 cm and a height of 20 cm. The spheres and the tank were filled with F-18 radionuclide solution with a total activity of 40.7 MBq (1.1 mCi) such that the uptake ratio of sphere to background was 3 to 1 for all spheres. The phantom was then scanned with a Gemini PET-CT scanner (Philips Medical System, Cleveland, Ohio). The reconstructed CT voxels were isotropic of size 0.6 mm. A slice of the CT image is shown in Figure 4(b). The PET FOV was 256 mm and a total of 18 frames were acquired each with acquisition time of 5 minutes. The acquired PET sinograms were reconstructed in STIR using 3D FBP as described in Section 2.4.2. The reconstructed PET voxel size was 2 × 2 × 3.15 mm^3^ in the *x*, *y*, and *z* directions, respectively. A slice of the PET image is shown in Figure 4(c). The PET PSF was measured in air using five F-18 point sources and the STIR parameters to reconstruct the PET images of the point sources were the same as those used to reconstruct the PET images of the sphere phantom. Gaussian fits were obtained in three orthogonal profiles to measure the FWHMs of PSF. The average FWHMs of the PSF were 7.23, 7.14, and 6.65 mm in the *x*, *y*, and *z* directions, respectively. Similar to the simulated sphere phantom, a total of 3 VOIs, that is, inner sphere volume, sphere walls, and the background volume were created, resulting in 3 × 3 weighting matrices to apply for all four PVC methods.

### 2.4. Image Analysis

#### 2.4.1. In-House IDL Software

An in-house image analysis software toolkit was developed in Interactive Data Language (IDL) version 8.1 (Research Systems Inc., Boulder, CO). This software was used to create the simulated PET images, display the 3D images, create and display VOIs, calculate the RSFs, implement the four PVC methods, and plot the results. The STIR routines for forward projection or reconstruction were called from the IDL software whenever they were needed (see below). For all PVC methods accuracy, precision, noise propagation characteristics, and the robustness in terms of PET-CT (or PET-MR) misregistration were evaluated.

The accuracy and precision were evaluated by calculating the mean and standard deviation of the recovery coefficient (RC) of a given PVC method using [27]

(5) $$\begin{array}{c} \text{RC}=\frac{\text{measured}\;\text{activity}\;\text{within}\;\text{VOI}}{\text{true}\;\text{activity}\;\text{within}\;\text{VOI}}. \end{array}$$

The ideal value for RC is unity; however, RC before PVC might be smaller or larger than unity if the VOI is hotter or colder than its surrounding background.

To evaluate the noise propagation characteristics of the PVC methods, noise magnification factors (NMFs) were calculated, that is, ratio of coefficient of variance after PVC to that before PVC [13]

(6) $$\begin{array}{c} \text{NMF}=\frac{dT_{i}/T_{i}}{dt_{i}/t_{i}}, \end{array}$$

where the *T*_*i*_ and *t*_*i*_ are as defined above for (1). The ideal value for NMF is unity. However, since PVC usually amplifies the noise, the value of the NMF is often greater than unity.

To evaluate robustness to misregistrations, the CT (or MR) mask images were shifted with respect to the PET images before performing PVC and the values of RC were calculated after the shifts. Misregistrations up to 10 mm were applied in the *x*, *y*, and *z* directions separately.

#### 2.4.2. STIR Forward Projection and Reconstruction

In order to perform the 3D forward projection and 3D reconstruction needed to implement the PVC methods in sinogram-space, routines from STIR release 2 [28] were used. These tasks require knowledge of the 3D PET detector geometry and STIR contains this information for a number of commercially available PET scanners including the Philips Allegro scanner used in this study. In order to call STIR routines, both image and sinogram data were converted to the interfile format [29] that is compatible with STIR.

In order to calculate RSFs for GTMo or sGTMo PVC methods in all three phantoms, VOI masks were forward projected and the sinograms were reconstructed using the STIR “fwdtest” and “fbp3drp” routines respectively. For all four PVC methods in all three phantoms, the PET images were reconstructed using the “fbp3drp” routine. This routine is based on a 3D FBP algorithm [30] for a given PET detector geometry. The measured sinogram data from the physical phantom scan needed to be corrected for detector gaps in the Philips Allegro scanner prior to reconstruction. Since STIR does not provide a routine for this task, an in-house gap filling algorithm based on a previous study [31] was implemented. The measured sinogram data was further corrected for attenuation and scatter before reconstruction. The attenuation correction was performed in STIR based on the measured CT images of the phantom. The scatter correction performed in STIR was based on the single scatter simulation algorithm [32].

## 3. Results

**Figure 5:**
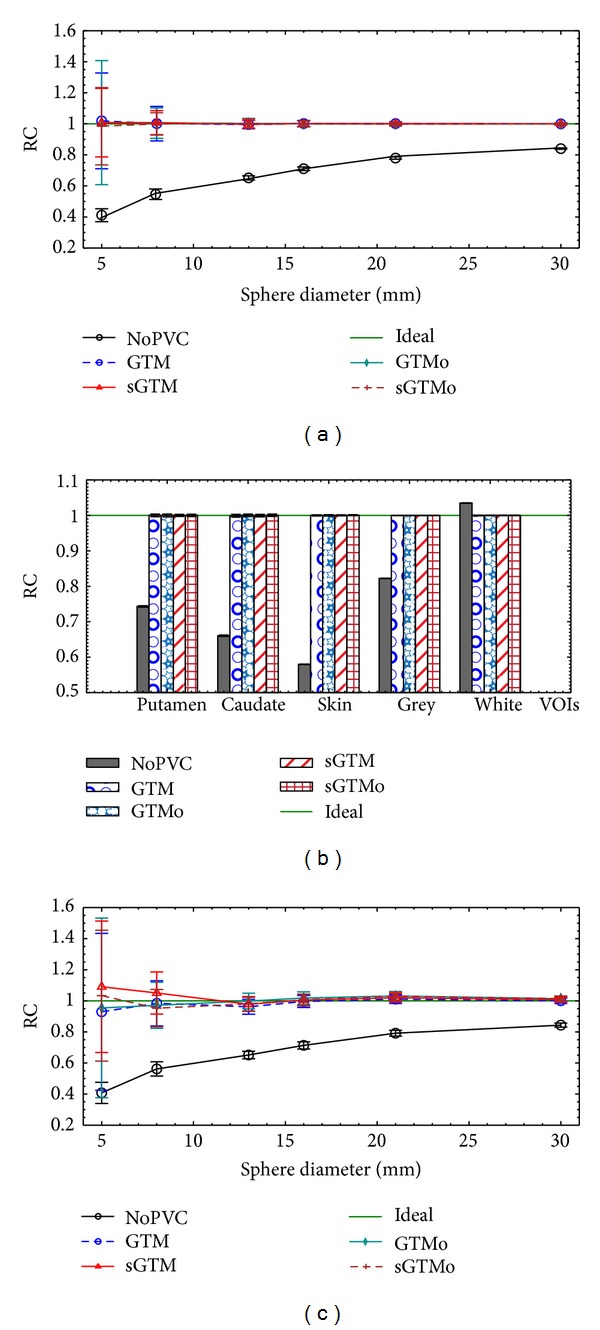
Plots of accuracy and precision of RC values using four PVC methods for the simulated sphere phantom (a), simulated brain phantom (b), and the physical sphere phantom (c). The error bars are standard deviations. The ideal values for RC are shown as green lines. RC: recovery coefficient, PVC: partial volume correction, GTM: geometric transfer matrix, sGTM: symmetric GTM, GTMo: GTM implemented in sinogram-space, sGTMo: sGTM implemented in sinogram-space, VOI: volume of interest, puta: putamen, caud: caudate nucleus, gry: grey matter, and wht: white matter.

Figure 5 shows the results for accuracy and precision of the RC values with different PVC methods and without using PVC. All RC values in Figure 5 are for the case without PET-CT (or PET-MR) misregistration. Figures 5(a), 5(b), and 5(c) are the results for simulated sphere, simulated brain, and the physical sphere phantoms, respectively. Figures 5(a) and 5(c) are for the inner sphere VOIs. In general, the corrected RCs for all brain and sphere VOIs and for all four PVC methods were within 5% of the ideal value. The only exceptions were for the smallest sphere size (5 mm diameter) for 2 PVC methods (GTM and sGTM) of the physical sphere phantom, although accuracy within 10% was still obtained even for this small object size. The variation in RC with image noise for the sGTM and sGTMo methods was less than that for the GTM and GTMo methods especially for smaller objects, as shown by the error bars for each method. The error bars for the simulated brain phantom in Figure 5(b) are significantly smaller than those for the sphere phantoms (Figures 5(a) and 5(c)). This effect is due to the fact that the brain VOIs had more voxels than the small sphere VOIs and noise is expected to affect smaller VOIs more than bigger VOIs.

**Figure 6:**
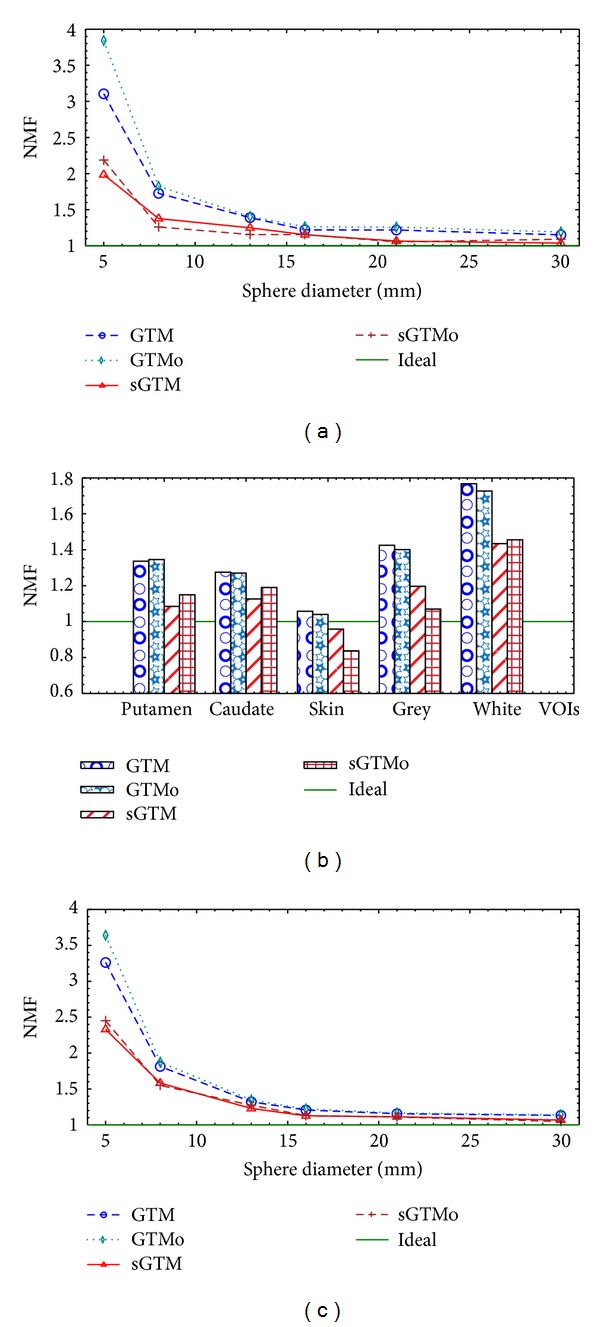
Noise propagation characteristics of four PVC methods expressed in terms of noise magnification factors (NMF) for the simulated sphere phantom (a), simulated brain phantom (b), and the physical sphere phantom (c). The ideal values for NMF are shown as green lines. The NMF values are smaller for sGTM and sGTMo compared to those of GTM and GTMo methods specially for smaller objects. NMF: noise magnification factor, GTM: geometric transfer matrix, sGTM: symmetric GTM, GTMo: GTM implemented in sinogram-space, sGTMo: sGTM implemented in sinogram-space, VOI: volume of interest, puta: putamen, caud: caudate nucleus, gry: grey matter, and wht: white matter.

Figure 6 shows the noise propagation plots characterized by the NMF values. The NMFs in Figure 6 are for the case with no errors in registration. The plots in Figures 6(a), 6(b), and 6(c) are the results for the simulated sphere phantom, simulated brain phantom, and the physical sphere phantom, respectively. Figures 6(a) and 6(c) are for the inner sphere VOIs. As expected, the NMF values for GTM were similar to those for GTMo method, and the values for sGTM were similar to those for sGTMo method. Moreover, the values of NMF for sGTMo were smaller than those of GTMo method indicating an improvement in noise propagation when the PVC matrix is symmetric, even when the PVC method is performed in the sinogram-space. The improvement in NMF is more pronounced for smaller objects.

**Figure 7:**
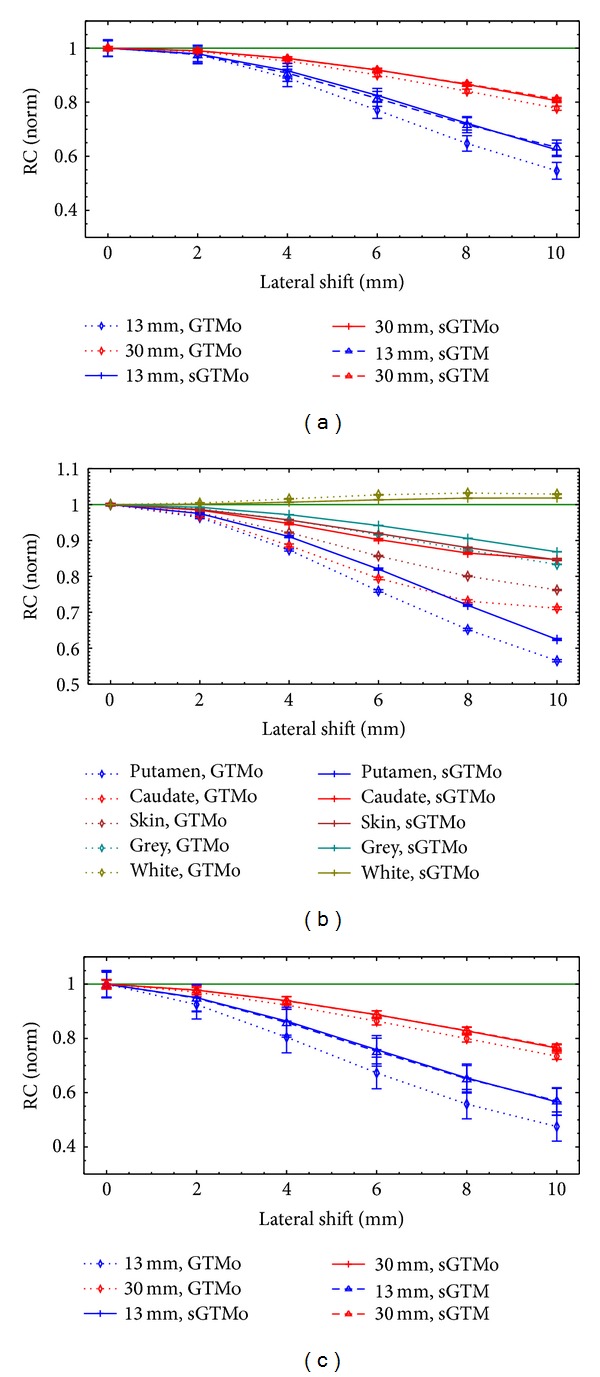
Comparison of robustness of the sGTMo to sGTM and GTMo methods in terms of registration errors. Normalized RCs are plotted as a function of registration error. The misregistration was applied in the lateral directions to (a) the simulated sphere phantom, (b) the simulated brain phantom, and (c) the physical sphere phantom. The error bars are standard deviations. The ideal values for RC are shown as green lines. RC: recovery coefficient, GTM: geometric transfer matrix, sGTM: symmetric GTM, GTMo: GTM implemented in sinogram-space, sGTMo: sGTM implemented in sinogram-space, VOI: volume of interest, puta: putamen, caud: caudate nucleus, gry: grey matter, and wht: white matter.

Figure 7 shows the normalized RC values when misregistration is applied between PET-CT (or between PET-MR) images. The plots in Figures 7(a), 7(b), and 7(c) are the results for the simulated sphere phantom, simulated brain phantom, and the physical sphere phantom, respectively. Figures 7(a) and 7(c) are for the inner sphere VOIs. Errors in registration were applied by shifting the CT (or MR) VOIs relative to the PET image before applying the PVC. All curves in Figure 7 are shown as a function of misregistration in the lateral (*x*) direction. The results of misregistration in other two directions (*y* and *z*) were similar (data not shown). The RC values in the figure were normalized to the RC values with zero misregistration. Data for one small (13 mm diameter) and one large (30 mm diameter) sphere is shown in Figures 7(a) and 7(c) to make the plots less cluttered. The results for other sphere sizes were similar (data not shown). The sGTM curves for the brain phantom and the GTM curves for all phantoms are not shown in Figure 7 in order to clarify other curves in the figure. However, as expected in all phantoms, the curve for GTM was close to that of GTMo and the curve for the sGTM was close to that of sGTMo. The results show that the symmetric PVC method is more robust than the nonsymmetric PVC method even if the PVC method is performed in the sinogram-space.

## 4. Discussion

In this study, the sGTMo PVC method, a sinogram implementation of sGTM, was implemented and validated, and its performance was compared to previously established region-based PVC methods. In order to test our two hypotheses, all four region-based PVC methods were applied to images of three different phantoms and their relative performance was evaluated in terms of accuracy, noise characteristics, and robustness with regard to PET-CT (or PET-MR) misregistrations. A discussion of how the results reflect upon the two hypotheses is presented below. In addition, situations where the sinogram implementation could be of interest are also discussed below.

### 4.1. Accuracy

The accuracy of the new sGTMo method is similar to all other region-based PVC methods. As shown in Figure 5, with no PVC on PET images, accuracy is lost as expected especially for smaller objects. However, using any of the four PVC methods and in the absence of registration errors, the accuracy of recovered uptake measurements will generally be within 5%.

### 4.2. Noise Characteristics

The results presented in Figure 6 show that the noise characteristic of the sGTMo method is similar to that of the sGTM method while it is improved compared to the GTMo method. This improvement is more pronounced for smaller objects as expected [25]. The precision (standard deviations) of the sGTMo method shown in Figure 5 is similar to that of the sGTM method while in general smaller than that of the GTMo method. Similarly, this is more notable for smaller objects. Better precision in the RC value translates to a better noise propagation characteristics when the weighting matrix is symmetric, regardless of its method of calculation, that is, image-based or sinogram-based.

The added noise for the simulations was uniform spatially across the image even though the uptake was not uniform within the image. This decision was based on a previous study [33] which showed that for the FBP algorithm, the noise is almost uniform even if the uptake is spatially nonuniform. The 25% noise level used in the simulations was the same noise level as obtained from the physical phantom. This corresponds to noisy PET images where the acquisition time is short (as for dynamic PET) or light filtration is applied during reconstruction. Note that the noise in the physical phantom experiments was inherently Poisson noise at the projection level, while, for simplicity, noise was added at the postreconstruction level for the phantom simulation experiments. The results in terms of correction sensitivity to noise level were similar for both physical experiments and simulations, suggesting that the differences in noise spectrum characteristics (i.e., one would expect a correlated image noise spectrum for noise added at the projection level) did not significantly change the interpretation of the results.

### 4.3. Robustness to Registration Errors

A practical PVC method for clinical applications requires robustness to PET-CT (or PET-MR) misregistration in order to preserve accuracy. Errors in registration may be due to patient movement during or between the two scans. The loss of accuracy due to misregistration has been regarded as a major source of error affecting region-based PVC methods [23]. Compared to other sources of error such as missegmentation or errors in PSF measurements, the misregistration has the strongest impact on accuracy and precision of uptake recovery [14]. The results in Figure 7 show that the sGTMo method is more robust than the GTMo method. Moreover, the sGTMo and sGTM methods are equally robust. The improvement in the robustness of sGTMo over GTMo is greater for smaller objects or tissue volumes. This was also the case when sGTM was compared to GTM in a previous study [25]. Thus, a symmetric implementation of the region-based PVC method is more robust to misregistration than the nonsymmetric implementation regardless of the image-based or sinogram-based approach.

It is interesting to point out that the registration errors do not affect the weighting matrices but only the *t* values on the right side of (1). As discussed previously [25], the improvement in robustness to misregisteration is due to the fact that the symmetric implementation samples the PET image (*t* values) by applying the RSFs with blurry boundaries rather than by utilizing the VOIs with sharp boundaries. Sampling the PET image with RSFs, in effect, takes advantage of resolution loss in PET. This implies that, for a given object size, the more resolution loss in PET, the more advantage in using the symmetric PVC (sGTM or sGTMo) than the nonsymmetric PVC (GTM or GTMo). Thus, the 3D implementation of the PVC (compared to 2D) is important to realize the advantage of improvement in robustness.

### 4.4. The Need for Sinogram Implementation

#### 4.4.1. Speed of Implementation

In the image-based PVC methods (GTM and sGTM), every VOI is convolved in 3D with the PET PSF to obtain the RSFs and thus the spillover information between the regions. On the other hand, in the sinogram-based PVC methods (GTMo and sGTMo), every VOI is forward projected and then reconstructed to get the same information on spillover. Unlike image-based PVC, the sinogram-based approach intrinsically accounts for local variations in the spatial resolution in non shift-invariant systems [5]. If the PSF is shift-invariant, the convolution step for the image-based PVC can be performed in Fourier domain using the fast Fourier transform (FFT) [34] which significantly speeds up the PVC implementation. Thus, the fast implementation of the image-based PVC is its major advantage over the sinogram-based PVC [23].

However, if the PSF is nonshift-invariant, the FFT cannot be used for convolution. Although in this case in principle, it is possible to perform the convolution in the image domain, in practice, this approach is not used for various reasons. For example, one needs a fully measured and characterized PSF for all points in the space. Moreover, it requires an extensive computational cost in terms of memory and speed. Thus, the advantage of a fast implementation for image-based PVC approach may be lost in nonshift-invariant systems. Nonshift-invariant behavior could be caused by detector parallax [35] which is more pronounced in small animal PET systems [36] due to small bore size. Another example is the head-dedicated PET systems in multislice configuration, where more than 40% difference exists between the axial PSF FWHM in the peripheral FOV compared to that of the central FOV [37].

Once the RSFs are calculated using either convolution or forward projection followed by reconstruction, the remaining implementation steps are based on (1) for all four PVC methods. This implies that the implementation of sGTM does not add any additional computational cost to the GTM method. Similarly, the implementation of sGTMo is just as fast as the GTMo method.

#### 4.4.2. Extension to Iterative Reconstructions

Due to their advantage over conventional analytic FBP algorithms, iterative reconstructions are commonly used today in the clinic for PET systems. Through repetitive forward projection and reconstruction techniques, iterative reconstructions model physical and statistical processes of photon production and detection more accurately and thus improve the image quality [38]. In terms of region-based PVCs, only a sinogram-based PVC method is readily extendable to iterative reconstructions [17]. In this method, the transfer matrix weighting factors are calculated through a perturbed forward projection and reconstruction technique, an approach which is not feasible for image-based PVC using the convolution technique. This is an advantage of the sinogram-based PVC over the image-based PVC.

## 5. Future Directions

The sinogram-space implementation of a region-based PVC method (GTMo) has been extended to iterative reconstruction algorithms [17]. These algorithms are now commonly used in the clinic due to their advantages over conventional FBP algorithms. An extension of the sGTMo method to iterative reconstructions is of great interest and is currently under investigation.

## 6. Conclusion

A sinogram-space implementation of the symmetric region-based PVC method (sGTMo) was implemented and validated using two 3D digital phantoms and one physical phantom. The performance of the sGTMo method was compared in terms of accuracy, noise characteristics, and robustness to registration errors to previously established region-based PVC methods, that is, sGTM and GTMo. The results confirm our two hypotheses that sGTMo method is similar in performance to the sGTM method while its performance is improved compared to the GTMo method.
